# A structured summary of a study protocol for a multi-center, randomized controlled trial (RCT) of COVID-19 prevention with Kampo medicines (Integrative Management in Japan for Epidemic Disease by prophylactic study: IMJEDI P1 study)

**DOI:** 10.1186/s13063-020-04939-2

**Published:** 2021-01-06

**Authors:** Takao Namiki, Shin Takayama, Ryutaro Arita, Tadashi Ishii, Mosaburo Kainuma, Toshiaki Makino, Masaru Mimura, Tetsuhiro Yoshino, Tatsuya Nogami, Makoto Arai, Juichi Sato, Koichiro Tanaka, Hajime Nakae, Hidetoshi Igari, Yoshihito Ozawa, Yuki Shiko, Yohei Kawasaki, Masahiko Nezu, Takashi Ito

**Affiliations:** 1 Department of Japanese-Oriental (Kampo) Medicine, Graduate School of Medicine, Chiba University, 1-8-1, Inohana, Chuo-ku, Chiba, 260-8670 Japan; 2 Department of Kampo Medicine, Tohoku University Hospital, 1-1, Seiryo-machi, Aoba-ku, Sendai, 980-8574 Japan; 3 Department of Education and Support for Regional Medicine, Tohoku University Hospital, 1- 1, Seiryo-machi, Aoba-ku, Sendai, 980-8574 Japan; 4 Department of Kampo and Integrative Medicine, Tohoku University Graduate School of Medicine, 1-2, Seiryo-machi, Aoba-ku, Sendai, 980-8575 Japan; 5 Community Medicine Education Unit, Graduate School of Medical Science, Kyushu University, 3-1-1 Maidashi, Higashi-ku, Fukuoka, 812-8582 Japan; 6 Department of Pharmacognosy, Graduate School of Pharmaceutical Sciences, Nagoya City University, 3-1 Tanabe-Dori, Mizuho-ku, Nagoya, 467-8603 Japan; 7 Department of Neuropsychiatry, Keio University School of Medicine, 35 Shinanomachi, Shinjuku-ku, Tokyo, 160-8582 Japan; 8 Center for Kampo Medicine, Keio University School of Medicine, 35 Shinanomachi, Shinjuku-ku, Tokyo, 160-8582 Japan; 9 Department of Kampo Medicine, Tokai University School of Medicine, 143 Shimokasuya, Isehara, Kanagawa 259-1193 Japan; 10 Department of General Medicine/Family & Community Medicine, Nagoya University Graduate School of Medicine, 65 Tsurumai-cho, Showa-ku, Nagoya, 466-8550 Japan; 11 Department of Traditional Medicine, Faculty of Medicine, Toho University, 5-21-16, Omorinishi, Ota-ku, Tokyo, 143-8540 Japan; 12 Department of Emergency and Critical Care Medicine, Akita University Graduate School of Medicine, 44-2 Hasunuma Hiroomote, Akita, 010-8543 Japan; 13 Department of Infectious Diseases, Chiba University Hospital, School of Medicine, Chiba University, 1-8-1, Inohana, Chuo-ku, Chiba, 260-8677 Japan; 14 Biostatistics Section, Clinical Research Center, Chiba University Hospital, 1-8-1 Inohana, Chuo-ku, Chiba, 260-8677 Japan; 15 Faculty of Nursing, Japanese Red Cross College of Nursing, Tokyo, 150-0012 Japan; 16 Akashi Clinic Kanda, 3-8, Kandaogawamachi, Chiyodaku, Tokyo, 101-0052 Japan

**Keywords:** COVID-19, randomized controlled trial, protocol, Kampo medicines, prophylactic study

## Abstract

**Objective:**

We aimed to test our hypothesis that traditional Japanese (Kampo) medicine, hochuekkito (Hochu-ekki-to: HET) has a preventive effect for the symptoms on COVID-19.

**Trial design:**

The study is designed as a multi-center, interventional, parallel-group, randomized (1:1 ratio), investigator sponsored, two-arm study.

**Participants:**

Six thousand participants will be recruited from healthy hospital workers in 7 Japanese University Hospitals.

Inclusion criteria:

1. Age from 20 to 75 years old at the time of registration

2. Asymptomatic and body temperature below 37°C at the time of registration

3. Capable of eating orally

Exclusion criteria:

1. Previous upper respiratory inflammation due to viral infection (including suspected COVID-19)

2. Taking immunosuppressants

3. Allergic to the Kampo medicines used in this study

4. History of hypokalaemia, severe hypertension, severe liver dysfunction, and interstitial pneumonia

5. Regularly taking other Kampo medicines

6. Pregnant or possibly pregnant

7. Participating in other research

8. Judged to be unsuitable for this study by the doctor in charge

**Intervention and comparator:**

Kampo group: participants receive HET in 9 tablets 2 times per day for 8 weeks.

Control group: participants receive placebo in the same dosage as the Intervention group - 9 tablets 2 times per day for 8 weeks. Placebo tablets are identical in appearance and package to HET. Taste of placebo is different from that of HET.

The Ohsugi Pharmaceutical Co. Ltd, Osaka, Japan manufactured the placebo and HET.

**Main outcomes:**

Primary outcome: Number of patients with a SARS-CoV-2 RNA by ploymerase chain reaction (PCR) positive result with at least one symptom (fever, cough, sputum, malaise, shortness of breath) during the 12-week study period (including the 4-week observation period after oral administration).

Secondary outcomes:

1. Period from infection to onset

2. Period from the appearance of symptoms to the disappearance of PCR positive

3. Number of days until the appearance or improvement of symptoms

4. Severe stage: presence of hospitalization

5. Shock stage: ICU management required for mechanical ventilation, shock vitals or failure of organ(s) other than lungs

Safety endpoints include numbness in the hands and/or feet, edema, skin rash or other allergic symptoms, and gastric discomfort.

**Randomisation:**

Patients are randomized (1:1 ratio) to each group using minimization implemented with the Electric data capture system (DATATRAK Enterprise Cloud), with balancing of the arms with age range (under 50 years of age or not) and having a history of risk factors for COVID-19 (cardiovascular disease, hypertension, diabetes, respiratory diseases).

**Blinding (masking):**

Only participants will be randomized.

**Numbers to be randomised (sample size):**

The main research hypothesis of this study is that Kampo medicines significantly prevent the onset of COVID-19. It is assumed that the infection rate before the administration of the drug under consideration will be 0% and that the incidence of COVID-19 thereafter will be 2- 3%, of which 70%-80% will show symptoms of COVID-19. Assuming that the pharmaceutical effect of the drug will be effective in 50% of patients and that the incidence rates in the placebo and drug groups will be 1.4%-2.4% and 0.7%-1.2%, respectively, the placebo is calculated at 2%, and the study drug at 1%. Since the frequency of verification is low and the number of cases will be large, we set a total of 10 analyses (9 interim analyses and a final analysis). Since the number of cases at the time of the final analysis will be 4,986 under the conditions of α = 0.05 and a power of 80% by the Peto method. We set at 600 cases in each interim analysis with an estimated dropout rate of 16.9%. Finally, the total number of cases is set to 6,000 with 3,000 in the placebo group and 3,000 in the HET group.

**Trial status:**

Protocol version 1.3 of October 23rd , 2020. Recruitment start (expected): December 1^st^, 2020. Recruitment finish (expected): December 31^st^, 2022.

**Trial registration:**

This trial is registered in the Japan Registry of Clinical Trials (jRCT) (jRCTs031200150) on 14 October 2020.

**Full protocol:**

The full protocol is attached as an additional file, accessible from the Trials website (Additional file 1). In the interest of expediting dissemination of this material, the familiar formatting has been eliminated; this Letter serves as a summary of the key elements of the full protocol.

**Supplementary Information:**

The online version contains supplementary material available at 10.1186/s13063-020-04939-2.

## Supplementary Information

**Additional file 1.** Full Study Protocol.

## Data Availability

Not applicable.

