# Supplementary material for: A structured summary of a study protocol for a multi-center, randomized controlled trial (RCT) of COVID-19 prevention with Kampo medicines (Integrative Management in Japan for Epidemic Disease by prophylactic study: IMJEDI P1 study)
Source: Trials. 2021 Jan 6;22:23. doi: 10.1186/s13063-020-04939-2 (PMC7787232; doi:10.1186/s13063-020-04939-2)
Supplement: Supplementary file 1 — Additional file 1. Full Study Protocol. [file 13063_2020_4939_MOESM1_ESM.docx]

試験実施計画書

COVID-19に対する漢方薬の予防に関するランダム化比較試験（IMJEDI-P１ Study）

第3相試験

研究責任者　並木隆雄（千葉大学医学部附属病院　和漢診療科　科長）

事務局　千葉大学医学部附属病院　和漢診療科

〒260-8677　千葉県千葉市中央区亥鼻1-8-1

（代表）043-222-7171

版数：1.3版

作成日：2020年10月23日

改訂履歴

| 作成日 | 版数 |
| --- | --- |
| 2020年6月23日  2020年8月11日 | 1.0版  1.1版 |
| 2020年9月 6日  2020年10月23日 | 1.2版  1.3版 |

本試験実施計画書における略語および用語の定義

| 略号・略記 | 英語表記 | 日本語表記、説明など |
| --- | --- | --- |
| MedDRA/J | Medical Dictionary for Regulatory Activities Terminology / J | ICH国際医薬用語集日本語版 |
| CTCAE | NCI-Common Terminology Criteria for Adverse Events |  |
| SARS-CoV-2 | Severe acute respiratory syndrome coronavirus 2 | 新型コロナウイルス |
| Novel Coronavirus | SARS-CoV-2 | 新型コロナウイルス |
| COVID-19 | Coronavirus Disease 2019 | 新型コロナウイルス感染症 |
| IMJEDI 19-P2 Study | Integrative management in Japan for epidemic disease; COVID 19- Prophylaxis Second study |  |

目次

0. 試験の概要 6

1. 緒言 8

1.1. 試験の背景 8

1.2. COVID-19に対する標準治療 8

1.3. 試験薬について 8

1.4. 試験薬の対象疾患に対する治療効果・試験成績 9

2. 試験の目的と必要性 9

3. 対象者 9

3.1. 選択基準 9

3.2. 除外基準 10

4. 被験者の同意 10

4.1. 同意文書及びその他の説明文書の作成並びに改訂 10

4.2. 同意取得の時期と方法 10

4.3. 被験者に対する説明事項 11

5. 試験の方法 12

5.1. 試験のデザイン 12

5.2. 試験のアウトライン 12

5.3. 目標被験者数と試験実施期間 13

5.4. 施設登録および症例登録・割付方法 13

5.4.1. 施設登録 13

5.4.2. 症例登録・割付方法 14

5.4.3. 割付調整因子 14

5.4.4. 症例登録先 14

5.5. 登録されなかった被験者の取り扱い 14

5.6. 投与スケジュールおよび投与量・投与方法 14

5.7. 減量基準 14

5.8. 休薬の基準 14

5.9 COVID19を疑う時 15

5.10. 個々の症例の中止基準 15

5.11. 併用薬 15

5.12. 併用禁止薬 15

5.13. 併用禁止療法 15

5.14. 後治療 15

5.15. 試験終了後の対応 16

6. 試験薬 16

6.1. 試験薬の概要 16

6.2. 試験薬の管理方法 16

6.3. 試験薬の配布方法 16

7. 観察・検査・評価項目、方法及び実施時期 17

7.1. 実施スケジュールと手順 17

7.1.1. アンケート調査 17

7.1.2. スクリーニング検査 18

7.1.3. 被験者の情報 18

7.1.4. 観察・検査・評価項目 18

7.1.4.1. 0週（Day0） 19

7.1.4.2. 2週（Day14） 19

7.1.4.3. 4週（Day 28） 19

7.1.4.4. 6週（Day42） 19

7.1.4.5. 8週（Day56） 20

7.1.4.6. 12週（Day84） 20

7.1.4.7. 中止時 20

8. 有害事象発生時の取扱い 20

8.1. 有害事象の定義 20

8.2. 有害事象発生時の被験者への対応 20

8.3. 報告の対象となる有害事象 20

8.4. 有害事象発生時の報告手順 21

8.5. 有害事象の評価に必要な記載内容 21

8.5.1. 有害事象の回復性と試験薬との因果関係 21

9. 重篤な有害事象発生時の取り扱い 21

9.1. 疾病等について 21

9.1.2. 疾病等（有害事象）の回復性と試験薬との因果関係 21

9.1.2.1. 疾病等（有害事象）発生時の対応 21

9.1.2.2. 疾病等（重篤な有害事象）発生時の対応 21

9.1.2.3. 疾病等（重篤な有害事象）報告の手順 21

9.2. 重篤な有害事象の定義 22

9.3. 報告の対象となる重篤な有害事象 23

10. 評価項目 23

10.1. 主要評価項目 23

10.2. 副次評価項目 23

10.3. 探索的評価項目 24

11. 統計学的事項 24

11.1. 解析対象集団 24

11.1.1. 最大の解析対象集団 (full analysis set：FAS) 24

11.1.2. 試験実施計画書に適合した対象集団 (per protocol set：PPS) 24

11.1.3. 安全性解析対象集団 25

11.2. 目標症例数と設定根拠 25

11.3. 症例の取り扱い 26

11.4. データの取り扱い 26

11.5. 統計解析項目および解析計画 26

11.5.1. 被験者背景の解析 26

11.5.1.1. 主要評価項目の解析 26

11.5.1.2. 副次評価項目の解析 26

11.5.1.3. 安全性の解析 26

11.5.1.4 中間解析 26

11.6. 独立データモニタリング委員会 27

11.7. 最終解析 27

12. 試験実施計画書の遵守および逸脱 27

13. 試験実施計画書、症例報告書又は解析計画に関する変更 27

13.1. 試験実施計画書および症例報告書の改訂 27

13.2. 統計解析計画の変更 28

14. 試験の中止、中断または終了 28

14.1. 試験全体での中止または中断の基準 28

14.2. 試験全体での中止又は中断する場合の手続き 28

14.3. 個々の実施医療機関での試験を中止または中断する場合の手続き 28

14.4. 試験の終了 29

15. データマネジメント 29

15.1. データ登録の方法及び管理方法 29

15.2. 症例報告書に直接記載され、かつ原資料(原データ)と解すべき資料の特定 29

16. 原資料及びその他の記録の保存 29

17. 試料等の保存及び他機関等の試料等の利用 30

18. 原資料の直接閲覧 30

19. 試験の品質管理及び品質保証 30

19.1. 品質管理 30

19.2. 品質保証 30

20.　 倫理 30

21. 被験者の秘密の保全 30

22.　認定臨床研究審査委員会 30

23.　健康被害補償及び保険 30

23.1. 健康被害の補償 30

23.2. 臨床研究保険（補償保険）への加入 31

23.3. 賠償保険への加入 31

24. 金銭の支払い 31

25. 研究資金および利益の衝突 31

1. 研究に関する情報公開 31
2. 結果の公表 32
   1. 公表の方法 32
   2. 公表についての取り決め 32

28. 参考資料・文献リスト 33

# **試験の概要**

| **試験課題名** | COVID-19に対する漢方薬の予防に関するランダム化比較試験（IMJEDI-P1 Study） |
| --- | --- |
| **試験の目的** | 医療従事者が補中益気湯エキスを内服することで、新型コロナウイルス感染後発症に至らない予防効果を検討する |
| **試験デザイン** | 検証的、単盲検試験、並行2群無作為化対照試験、多施設共同 |
| **フェーズ** | 第3相 |
| **被験薬** | 一般名：ジュンコウ補中益気湯（FCエキス錠剤　医療用）  剤形： 内用剤  保存条件：室温保存 |
| **選択基準** | COVID-19に未感染の医療関係者（無症状）において以下のすべての条件に該当する患者を対象とする。   1. 年齢：20歳以上75歳までの方（登録時） 2. 無症状かつ体温37.0度以下（登録時） 3. 経口摂取可能なもの |
| **除外基準** | 以下のいずれかの条件に該当する者は対象としない。   1. すでに新型コロナウイルスを含むウイルスによる上気道炎を発症（疑いも含む）している者 2. 免疫抑制剤を内服している者 3. 漢方薬に対するアレルギーを有する者 4. 低カリウム血症・重度の高血圧、重篤な肝機能障害、間質性肺炎の既往のある方感染（自己申告に基づく）から発症までの期間 5. 他の漢方薬を定期的に内服している者 6. 妊娠中または妊娠の可能性のある者 7. 他の研究に参加中の者 8. その他担当医師が本研究の対象として好ましくないと判断した者 |
| **評価項目** | １**．主要評価項目**  症状を有するCOVID-19のPCR陽性者数  （症状とは37.5度以上の発熱、倦怠感、咳嗽、咽頭痛、嘔吐、下痢、呼吸困難、味覚・嗅覚異常などの感冒様症状を指す）。 ２．**副次評価項目** **有効性の副次評価項目**  ・感染（自己申告に基づく）から発症までの期間、  ・症状出現からPCR陰性化までの期間  ・症状出現か症状の改善までの日数  ・酸素吸入を有する入院（重症）の有無  ・重篤（機械換気を要する、ショックバイタル、肺以外の臓器不全にてICU 管理が必要）  **安全性の副次評価項目**  有害事象の発生頻度  ３．**探索的評価項目**  千葉大学の患者のうち先着順で選んだ60名に対し補中益気湯の有効時のメカニズムを探索するために、特殊検査として以下を内服期間中経時的に行う  自然免疫検査（当日）： Nk細胞活性、CD 16/CD56、CD4/CD8陽性細胞数  獲得免疫検査（後日）TNFα、ILシリーズ（IL-2、IL-4、IL-5、IL-6、IL-17濃度など）およびメタボロミクス解析（選択検体のみ） |
| **試験方法** | 適格性を審査後、1週間程度の観察期のあと、登録時に中央方式でジュンコウ補中益気湯エキス錠剤または偽薬に割り付けの錠剤にいずれか18錠を1日2分割して食前または食間での経口投与を8週間（56日）継続する。その間のCOVID-19の発症状況を観察する。  千葉大で行う症例60名については承諾の得られた被験者は、投与前と8週まで2週ごとの採血をおこない、免疫系の変化を検討する。 |
| **追加治療：治療不応例に対する治療選択** | 該当なし |
| **目標被験者数** | 6000例 |
| **試験実施期間** | 試験実施期間：3年9か月(2020年承認後-2024年3月31日)  症例登録期間：2年6カ月 (2020年承認後- 2022年12月31日) |
| **試験施設数** | 3 |
| **倫理指針** | 本試験の実施に際しては「ヘルシンキ宣言」に基づく倫理的原則、及び臨床研究法、その他の関連する規制要件を遵守するものとする。 |
| **認定臨床研究審査委員会** | 本試験の実施に先立ち、実施医療機関の認定臨床研究審査委員会は、本試験の倫理的、科学的及び医学的妥当性を審査する。本試験は、認定臨床研究審査委員会の承認を得た後に実施する。また、認定臨床研究審査委員会は厚生労働省令で定めるところにより、定期的に特定臨床研究の実施状況について報告を受け、当該特定臨床研究の継続的の適否について意見を述べる。 |

# **緒言**

## **試験の背景**

2019年末から発生した新型コロナウイルス　SARS-CoV-2（Severe acute respiratory syndrome coronavirus 2）は世界中に蔓延し、感染者数の急激な増加と重症者・死亡者の対応に追われ、医療従事者の負担が日毎に重くなっている。濃厚接触をする医療従事者は新型コロナウイルスによる呼吸器疾患（Coronavirus disease 2019, COVID-19）により、海外では医療従事者の感染事故が生じ、その中には死亡例などの不幸の転機になるものが多数報告されているが、これは本邦でも今後十分に起こりうる事態である。また、そのような感染症にさらされている医療現場では、さらに医療関係者が自己への不安を訴える例など多くの海外での報告がある。^1-2）^新型コロナウイルスに対する予防法としては、手洗いによる飛沫や接触感染の標準予防策を実施することだけである。一方、ワクチン開発と獲得免疫による新型コロナウイルス根本治療の開発まで、安全性を担保するための臨床試験が必要なため、最低1-2年はかかると推測されている。

免疫には、大きく分けて自然免疫系と獲得免疫系が存在する。身体にとって、まず未遭遇のウイルスなどの微生物に免疫反応を示す必要がある場合は、前者の自然免疫系が動員される。この自然免疫系の賦活をすれば、外界からの新生物に対しての最初の防御強化になると推測される。自然免疫系を賦活するには、良質な食事・適切な睡眠・ストレスのコントロールなどの生活習慣の改善に加え、漢方薬での報告が散見される。特に補中益気湯は自然免疫系への影響に対する報告が多い漢方薬の一つである。

新型コロナウイルスが発症した中国では、中国での感染マニュアルの最後に伝統医学の治療も書かれている^3)^。多くの症例に伝統医学を併用しCOVID-19に対する有効性の報告がされている。中国語での論文であるため詳細がわからない点や評価方法の問題点がある。中国伝統医学の治験は行われているが、WHO-China report^4)^には「ただし、治験薬の評価には、現実的な適格性基準と患者の適切な層別化を備えた、十分な能力を備えたランダム化比較試験が必要である。 中国国内および中国外で実施している研究の間にはある程度の調整があることが重要である」との指摘がある。日本国内で使用できる伝統薬である漢方薬は、中国伝統医学とルーツは同じであるが、多くの点で異なっており、中国の経験をそのまま日本に持ち込めない。中国での治療に用いた伝統医学処方は日本で使用できる漢方薬とは構成が異なっているなどの問題点がある。したがって、中国の使用経験は参考になるが、日本独自の漢方薬を高いレベルで~~の~~再評価する必要がある。

## **COVID-19に対する標準治療**

2020年5月20日時点でCOVID-19に対する標準治療は確立しておらず、まだ、ワクチンなどの開発途上であるため、完全な予防方法はない。なお、現時点ではワクチン開発までに1－2年以上かかると思われる。

## **試験薬について**

日本においては、補中益気湯は倦怠感・食欲不振・感冒に適応となっている漢方エキス薬であり、過去の研究からウイルス感染予防、感冒回数減少効果が報告されている^5）^。動物実験でも正常動物において、NK細胞活性の上昇やCD4/CD8比の上昇がいずれも有意であった^6）^。ヒトでもNK細胞活性の上昇を認めた^7）^。ウイルス疾患からの予防効果さらに病態モデルでは、インフルエンザ（N1H1）のを経鼻摂取したマウスに、接種前から投与を始め、接種後4日目まで投与したところ、用量によらず有意な生存日数の延長を認めた。これらのことから、補中益気湯において、ウイルス疾患の予防の可能性が考えられる。本研究は参加者が補中益気湯エキスを内服することで、COVID‐19を予防できるかどうかを検討する。

なお、基礎研究で各種サイトカインは有意に低下した^9）^。このことは、補中益気湯がサイトカインストームなどの急激な悪化状態を抑える可能性を示唆する可能性があるため、発症後に内服を続けた場合は、重症化や重篤化への影響も検討する。試験の際に、協力を得られた一部の被験者に対しては、自覚症状、身体所見、採血により免疫の賦活の状態と感染の関係について、後ろ向きに検討することで免疫機能に対するメカニズムを探索的に検討する。安全性については、甘草が含まれるため、偽アルドステロン症が発症する可能性や薬剤性の肝障害などの報告があるので、試験期間中に経過観察を十分に行う。

## **試験薬の対象疾患に対する治療効果・試験成績**

補中益気湯のCOVID-19に対する直接の効果の報告はない。

# **試験の目的と必要性**

（目的と研究仮説）

医療従事者を対象に補中益気湯エキスを内服することで、新型コロナウイルス感染後発症に至らない予防効果を検討する。

COVID-19は無症状陽性が多いことや日本では、PCR検査が広くいきわたっていないため、背景としてすでに既往または、現在感染している方がわからないため、背景を均一化する目的で2群間の比較ではランダム化比較試験が必須となる。ランダム化比較試験は客観性が高い。しかし、偽薬がなく2か月の経過観察はコントロール群では難しい。そこで、漢方薬の中で比較的偽薬が作りやすい錠剤でのランダム化を検討したところ、製薬会社1社が、偽薬も含めて薬物の供出に応じていただけたため、本検討は可能となった。仮説としては、漢方薬の投与により、免疫賦活のため感染が成立しづらくなると考えられる。一方、PCR検査が被験者の全員にできないことから、主要評価項目として内服前後での感染に関してPCR陽性の有無での鑑別は難しいため、症状を有するCOVID-19のPCR陽性者数を主要評価項目とした。なお、症状とは37.5度以上の発熱、倦怠感、咳嗽、咽頭痛、嘔吐、下痢、呼吸困難、味覚・嗅覚異常などの感冒様症状を指すこととした。

また、副次評価項目は発病後の経過が偽薬群と比較による改善の有無にした。感染（自己申告に基づく）から発症までの期間、発症から症状の消失までの期間、発症からPCR陰性化までの期間、さらに酸素化の低下による重症化は感染者の20％程度に起こると考えられている。その率の低下をとした。さらに全体の2-3％に生じるといわれている重篤（機械換気を要する、ショックバイタル、肺以外の臓器不全にてICU 管理が必要）も併せて評価することとした。

（本試験の必要性、臨床上の意義）

標準的予防策を取っていても、感染する可能性のある医療関係者に対して、本薬の投与で少しでも感染リスクを減らせる可能性があった場合

1. 感染による医療関係者の隔離によって、医療関係者の数の減少を食い止められることができる。
2. このことにより、未感染の医療関係者の負担の間接的な軽減が図れることができる。
3. 感染が減れば、そのためにある一定程度の重篤症化する医療関係者を直接減らせることができる。
4. 予防ができる処方が見つかることで、COVID-19感染の心理的負担の軽減が図れることができる。

などの効果が見込まれる。

# **対象患者**

## **選択基準**

対象：COVID-19に未感染の医療関係者（無症状）

1. 年齢：20歳以上75歳までの方（登録時）
2. 無症状かつ体温37.0度以下（登録時）
3. 経口摂取可能なもの

インターネット経由での承諾をする者に関しては以下も併せて選択基準とする

1. パソコン、スマートフォン、タブレットを用いて、インターネット使用環境
   下にある者
2. 日本語による説明を理解し、本人の自由意思によるオンライン上の同意が得
   られた者

【設定の根拠】

1)-4)：対象患者の安全性及び倫理性を考慮し、信頼性のあるデータを得るために設定した。

A)B)はインターネット使用環境下での同意のために設定した。

## **除外基準**

**（有効性評価に影響を及ぼすための除外）**

1. すでに新型コロナウイルスを含むウイルスによる上気道炎を発症（疑いも含む）した者
2. 免疫抑制剤を内服している者

**（安全性評価に影響を及ぼすための除外）**

1. 漢方薬に対するアレルギーを有する者
2. 低カリウム血症、重度の高血圧症、重篤な肝機能障害、間質性肺炎の既往のある者
3. 他の漢方薬を定期的に内服している者（低カリウム症の誘発）
4. 妊娠中または妊娠の可能性のある者

**（全般的理由からの除外）**

1. 他の研究に参加中の者
2. その他担当医師が本研究の対象として好ましくないと判断した者

【設定根拠】

1)-2)5）：本試験の有効性の評価に影響を与えると考えられるため、設定した。

3)‐6）：対象患者の安全性を考慮し、設定した。

# **被験者の同意**

## **同意文書及びその他の説明文書の作成並びに改訂**

試験責任医師は、被験者から試験参加の直接面談またはインターネット上での同意を得るために用いる同意文書及びその他の説明文書を得るための文書を可能な限り平易な表現で作成する。また、同意文書及びその他の説明文書を改訂する必要があると認めた場合は、これらを改訂する。

　試験責任医師は、作成又は改訂された同意文書及びその他の説明文書を認定臨床研究審査委員会に提出し、その承認を得る。

## **同意取得の時期と方法**

1. **アンケート調査への同意の取得**

アンケート調査への参加を希望する被験者より、アンケート調査項目回答に先たち調査様式上で、アンケート調査への参加同意を習得する。

1. **試験参加の同意の取得**

**直接面談での同意説明が困難な場合**

試験責任医師又は試験分担医師は、同意文書及びその他の説明文書を参加条件について満たしたことが確定した段階で提示する。添付文書として被験者に提示し、「4.3被験者に対する説明事項」に示す内容について十分説明を行う。必要であればインターネット上の動画（YouTube）を利用して説明をする。被験者が試験の内容を理解したことを確認した上で、試験開始前(スクリーニング)検査や問診を実施するまでに文書を提示する。これを読んで理解し、自由意思で参加することに同意した者を本研究にエントリーさせる。

**面談での同意説明が可能な場合**

試験責任医師又は試験分担医師は、同意文書及びその他の説明文書を被験者に手渡し、「4.3被験者に対する説明事項」に示す内容について十分な説明を行う。
被験者が試験の内容を良く理解したことを確認した上で、試験開始前(スクリーニング)検査を実施するまでに文書で自由意思による同意を取得する。

1. **試験参加の同意書への記入方法および説明文書の交付**

**直接面談での同意説明が困難な場合**

口頭でのインフォームド・コンセントではなく、インターネット経由での添付文書で同意書を被験者に提示する。印刷した同意書へ記載してもらう形でのインフォームド・コンセントの手続を行う。被験者は同意書に署名し、同意した日付を記入する。同意書は、和漢診療科外来受付に設置した返却箱に提出する。提出後、試験責任医師又は試験分担医師が記名捺印又は署名し、確認した日付を記入する。その同意書の写しを被験者にすみやかに交付することで同意の確認とする（原本は事務局で預かる）。

**面談での同意説明が可能な場合**

　被験者の同意に際しては、説明を行った試験責任医師又は試験分担医師が記名捺印又は署名し、説明した日付を記入する。被験者は同意書に署名し、同意した日付を記入する。同意を得た後、説明文書及び同意書の写しを被験者に交付する。

1. **説明文書改訂時**

試験責任医師又は試験分担医師は、被験者の同意に関連し得る新たな情報の入手などにより同意文書及びその他の説明文書を改訂した場合、被験者に対して改訂された同意文書及びその他の説明文書を用いて改めて説明し、試験への参加継続について文書で同意を取得する。

　インターネット経由での参加同意をした方には改訂された同意文書及びその他の説明文書を用

　いて改めて、添付文書として被験者に提示し、同意書をもらう形での本試験への参加継続に

ついてのインフォームド・コンセントの手続を行う。

## **被験者に対する説明事項**

臨床研究の名称、本臨床研究の実施について研究実施医療機関の管理者の承認を受けている旨及び厚生労働大臣に実施計画を提出している旨

研究実施医療機関の名称並びに研究責任医師の氏名及び職名、連絡先、試験分担医師の氏名、職名（研究代表医師の氏名及び職名、連絡先、他の研究実施医療機関の名称並びに当該研究実施医療機関の研究責任医師の氏名及び職名、連絡先を含む）

本臨床研究の対象者として選定された理由

本臨床研究の目的

臨床試験の方法(臨床試験の試験的側面、被験者の選択基準及び各投与群に割付られる確率を含む)

被験者の臨床試験への参加予定期間

臨床試験に参加する予定の被験者数

本臨床研究の実施により予期される利益及び不利益

本臨床研究への参加を拒否することは任意である旨

同意の撤回に関する事項

1. 臨床試験への参加は被験者の自由意思によるものであり、被験者は臨床試験への参加を随時拒否又は撤回することができること。また、拒否・撤回によって被験者が不利な扱いを受けること、臨床試験に参加しない場合にうけるべき利益を失うことはないこと

本臨床研究に関する情報公開の方法

本臨床研究の対象者の求めに対して、研究計画書その他の本臨床研究の実施に関する資料を入手又は、閲覧できる旨及びその入手又は閲覧の方法

本臨床研究の対象者の個人情報の保護に関する事項

資料等の保管及び廃棄の方法

本臨床研究に対する利益相反管理に関する状況

苦情及び相談窓口、問い合わせへの対応に関する体制

本臨床研究の実施に係る費用に関する事項

本臨床研究の実施による健康被害に対する補償及び医療の提供に関する事項

本臨床研究の参加継続について被験者の意思に影響を与える可能性のある情報が得られた場合には速やかに被験者に伝えられること

1. 本臨床研究の参加を中止させる場合の条件又は理由

被験者が守るべき事項

その他本臨床研究の実施に関し必要な事項

知的財産

# **試験の方法**

## **試験のデザイン**

本試験は検証的、単盲検試験、並行2群間無作為化試験、多施設共同試験である。

## **試験のアウトライン**

対象者において、アンケートで選択基準または除外基準を満たした者について、試験に関しての説明後承諾書を取得する。中央方式で割り付け因子（年齢と合併症の2つ）を考慮して、ランダム化して登録する。

ジュンコウ補中益気湯エキス錠剤（実薬群）または、包装により識別不可能な偽薬群のいずれかに割りつけて、各群とも18錠を1日2分割して食前または食間での経口投与を8週間（56日）継続する。その期間中は、日記（紙または電子的）に、下記を記録する。

1. 自覚症状(感冒症状を中心に身体症状)
2. バイタルサイン(体温・必要時呼吸数/SpO_2、_浮腫時血圧測定)
3. 体重測定

紙による日記（添付書類）は毎週特定日に回収してする。日記において異常な自覚・他覚所見（浮腫と内服後の高血圧）が見つかった場合および自己申告での希望時は医師の診察を受ける。

電子的な参加者データ収集は、Microsoft formsを用いて毎日行う。その際参加者は各人ごとの登録番号を用いて送付するため、プライバシーは担保される。それらの情報により、参加者の健康状態を把握する。自己申告による受診希望や上記情報により副作用が発見された場合は医師の診察を受けられる体制とする。

安全性の確認のため、投与終了後最大4週間の追跡期間を設ける。COVID-19の症状やCOVID-19のPCRが陽性でも、原則内服を中止しない。何らかの理由で、内服を中止した場合は、試験期間に含めないが、試験終了後の追跡期間最大4週間は上記に準じて設ける。

従って、本研究に参加された場合の参加者の予定参加期間は、前観察期間１週間、研究薬投与期間８週間、後観察期間４週間の計１3週間となる。

## なお、先着順で採血に同意した被験者で承諾が取れるもの60名については、後述の2週間ごとの採血を行う。


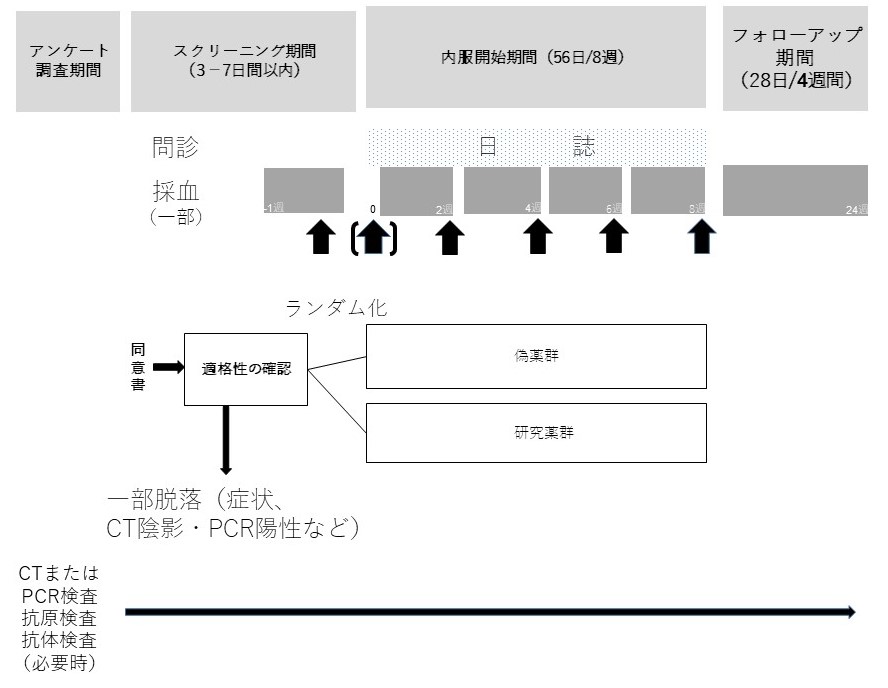


## **目標被験者数と試験実施期間**

目標被験者数：6000例

## 試験実施期間： 3年9か月 (2020年承認後 -2024年3月31日)

症例登録期間： 2年6か月 (2020年承認後- 2022年12月31日)

目標被験者を、1回ごと600名で10回（Step1-10）に分けて参加者を募集する。また、感染率が結果に影響するため、市中での感染率おおむね2％以上になった場合に試験を開始する（対象病院周囲でのPCR検査、抗原検査、抗体検査などや発症の程度から推測する）。

現時点での予定（感染の状況により変更することもある）

2020-21年シーズンStep1(千葉大学)、Step2(東北大学)、Step3(九州大学)、Step4(千葉大学)

2021-22年シーズンStep5(東北大学)、Step6(九州大学)、Step7(千葉大学)、

　2022-23年シーズンStep8(東北大学)、Step9(九州大学)、Step10(千葉大学)

## **施設登録および症例登録**

施設登録および症例登録は、千葉大学医学部附属病院データセンターにおける中央登録制とする。施設登録が完了した施設から症例登録が可能となる。施設登録はFAX、症例登録はWebにより、以下の手順で行う。なお、症例登録については各施設で被験者識別コードを発番し対応表を作成する。

### **施設登録**

1) 各参加施設の試験責任医師は、当該の施設の認定臨床研究審査委員会等での承認が得られた後、臨床研究審査委員会等の承認通知書の写し及び施設登録依頼書をデータセンターにFAXまたは持参する。

2) データセンターは施設登録を行い、施設登録完了通知書の写しを試験責任医師に送付する。

### **症例登録・割付方法**

1. 登録は、原則として同意取得後7日以内に行うこととする。
2. 試験責任医師または試験分担医師は、文書による同意を取得し、スクリーニング検査の結果、被験者が選択基準を満たし、除外基準に抵触していないことを確認する。本試験での被験者の登録は、「症例登録票」をFAX、持参もしくはWeb upにより行う。
3. 試験責任医師または試験分担医師は、下記のWebサイトにアクセスし、症例登録のページに必要な情報を入力し、症例登録を行う。詳細は別途作成する被験者症例登録入力マニュアルを参照する。症例登録に必要な情報を入力後、割付を行う。割付結果は、症例登録Web画面に表示される。研究責任医師または研究分担医師は、割付結果に従いプロトコル治療を開始する。一度登録された患者は登録取り消し（データベースから抹消）はなされない。重複登録の場合は、いかなる場合も初回の登録情報（登録番号）・割付群を採用する。研究責任医師または研究分担医師は誤登録・重複登録が判明した際には速やかにデータセンターに連絡する。

※ 研究責任医師又は研究分担医師は、被験者の登録・割付がなされるまで試験薬を処方・投与してはならない。

### **割付調整因子**

### 割付調整因子は、自己申告に基づいた年齢（50歳未満、50歳以上）、合併症の有無（心疾患、高血圧、糖尿病、呼吸器疾患）の2因子とする

### **症例登録先**

千葉大学医学部附属病院 データセンター

受付時間：午前9時～午後5時

TEL：043-221-7171（内線：6593）

FAX：043-226-2644

## **登録されなかった被験者の取り扱い**

登録において、割付前に不適格などの何らかの理由で割付が行われなかった場合は、登録されなかった被験者となり、試験の登録症例には含めない。試験責任医師又は試験分担医師は、当該被験者に本試験への登録が不可である旨を説明する。

## **投与スケジュールおよび投与量・投与方法**

ジュンコウ補中益気湯FCエキス錠剤（医療用）18錠を1日2回に分けて食前または食間に経口投与する。

## **減量基準**

減量基準は設定しない。

## **休薬の基準**

以下の基準に該当した場合、試験責任医師又は試験分担医師は試験薬投与を一旦中止する。

ただし、検査スケジュールに沿った検査・評価は継続して実施する。

1. 試験責任医師又は試験分担医師が試験薬の投与を不適当と認めた場合。
2. 試験責任医師又は試験分担医師が試験薬の投与中止を必要と認めた場合。
3. 血清カリウム値が各施設の正常下限以下になった場合。
4. 7日以上続く血圧上昇が現れ、試験責任医師又は試験分担医師が試験薬の投与中止を必要と判断された場合。
5. その他、有害事象の発現等により試験責任医師又は試験分担医師が試験薬の休薬を必要と判断した場合。

## **COVID-19を疑う時**

内服期間または内服終了後の観察期間に感冒様症状が出現し、1日以上継続した時は、試験事務局に連絡をもらうとともに、感染治療部に連絡するように伝える。COVID-19の診断は、感染治療部の医師の診断により決定する。なお、症状とは37.5度以上の発熱、倦怠感、咳嗽、咽頭痛、嘔吐、下痢、呼吸困難、味覚・嗅覚異常などの感冒様症状を指す。

感染後においても、原則、補中益気湯の内服は継続する（被検者の中止希望がある場合を除く）。

## **個々の症例の中止基準**

以下の基準に該当した場合、試験責任医師又は試験分担医師は試験薬投与を中止する。ただし、検査スケジュールに沿った検査・評価は継続して実施する。

1. 試験薬の休薬後も有害事象が持続し、試験責任医師又は試験分担医師により中止が必要と判断された場合。
2. 被験者からの中止の申し出があった場合。
3. 研究代表者または研究調整委員会により中止の決定が判断された場合。
4. SpO2が90%以下またはCOVID-19に対する他の治療が開始され本薬剤の中止を指示された場合。
5. その他、試験責任医師又は試験分担医師により被験者の試験継続が不可能と判断された場合

試験が中止された場合の「中止日」は、中止の理由となる事象が発現した日ではなく、試験責任医師又は試験分担医師が中止を判断した日とする。

注意1：試験担当医師は何らかの理由で試験継続が不可能と判断した場合には、試験薬の投与を中止する。中止･脱落の日付･時期、中止･脱落の理由、経過をカルテならびに症例報告書に記載するとともに、中止･脱落時点で必要な検査を行い、有効性･安全性の評価を行う。

注意2：試験薬投与開始後に同意の撤回があった場合は、試験薬の効果不発揮あるいは有害事象によるものか、あるいは偶発的事象（転居等）によるものかをできるだけ明らかにし、有効性・安全性評価の対象となる症例としての採否の参考となるように記録する。

## **併用薬**

該当なし

## **併用禁止薬**

1. 免疫抑制剤
2. 定期的に内服している他の漢方薬（屯用を除く）
3. COVID‐19に対する治療薬（または治療候補で治験中のもの）

## **併用禁止療法**

　該当なし

## **後治療**

　該当なし

## **試験終了後の対応**

試験終了後に追跡期間を4週間設ける。参加者がその間に当該薬の影響が疑われる症状・病気が生じた場合は、最善の診断及び治療を受けることができるよう努める。

# **試験薬**

- 1. **試験薬の概要**

ジュンコウ補中益気湯エキス錠剤（実薬群）または、包装により識別不可能な偽薬群のいずれかに割りつけて、各群とも18錠を1日2分割して食前または食間での経口投与を8週間（56日）継続する。その期間中は、日記（紙または電子的）に、下記を記録する。

試験薬の詳細及び取り扱いに関しては、添付文書参照。本試験で使用する試験薬は以下のとおりである。

一般名：ジュンコウ補中益気湯FCエキス錠剤

剤形：錠剤

含有量：本剤18錠に下記の割合の混合生薬の乾燥エキス4.9gを含有する。

　　　　日局オウギ　4.0g　日局タイソウ　2.0g　日局ビャクジュツ　4.0g　日局チンピ　2.0g

　　　　日局ニンジン　4.0g　日局カンゾウ　1.5g　日局トウキ　3.0g　日局ショウマ　1.0g

　　　　日局サイコ　2.0g　日局ショウキョウ　0.5g　日局ビャクジュツ 4.0g

性状：色　淡褐色

包装：包装

貯法：遮光

保存条件：室温保存

副作用：本剤は使用成績調査などの副作用発現頻度が明瞭となる調査を実施していないため、発言頻度は不明である。

重大な副作用

- - - 1. 間質性肺炎
      2. 偽アルドステロン症
      3. ミオパチー
      4. 肝機能障害、黄疸
      5. その他の副作用：過敏症（発疹、蕁麻疹）、消化器（食欲不振、胃部不快感、悪心、下痢等）

偽薬（プラセボ）

際系は：錠剤

含有物：乳糖を主体として固め、実薬を同じ形状にした。薬効を有しない錠剤。

性状：色　淡褐色

包装：包装は実薬と区別のつかない同一の包装とし

貯法：遮光

保存条件：室温保存

- 1. **試験薬の管理方法**

保存条件：室温保存

試験薬は大杉製薬株式会社より提供される。

1. **試験薬の配布方法**

適格性が判断され被験者の同意を得たのち、試験薬は和漢診療科外来で配布される。

# **観察・検査・評価項目、方法及び実施時期**

**7.1実施スケジュールと手順**

| 時期  項目 | アン  ケート  調査  期間 | スクリーニング  期間 | 内服期間 | | | | | 観察期間 | 中止時 |
| --- | --- | --- | --- | --- | --- | --- | --- | --- | --- |
|  |  |  | 0週  Day0 | 2週  Day14 | 4週  Day28 | 6週  Day42 | 8週  Day56 | 12週  Day84 |  |
| 許容範囲  (日) |  | 3-7 | 0 | ±2 | ±7 | ±７ | ±７ | ±14 |  |
| 同意取得 |  | ● |  |  |  |  |  |  |  |
| 被験者背景C | ●C |  |  |  |  |  |  |  |  |
| COVID-19症状の有無 |  | ● | ○ | ● | ● | ● | ● | ● |  |
| COVID-19治療の有無 |  | ● | ● | | | | | ● |  |
| 血液検査（任意）a  (60名にのみ) |  | ● | ○ｄ | ● | ● | ● | ● |  |  |
| 有害事象の観察b |  |  | ● | | | | | ● | ● |
| 体温測定 | ● |  | ●（毎日測定） | | | | | ● |  |
| 体重測定c | ● |  |  | ● | ● | ● | ● | ● | ● |
| SpO2、 | ● |  | 〇 | | | | |  |  |
| 自他覚  症状観察（アプリ）e | ● | ○ | ●（毎日送信） | | | | | ● | ● |
| 自他覚  症状観察（日誌）e | ● | 〇 | ●（毎日記入、一週間ごとに事務局に提出） | | | | |  |  |
| 併用薬の調査 | ● |  |  | | | | |  |  |

●印は必須項目、〇印は必要時に行う項目

症状発生時は感染症内科へ受診し、PCR検査を受けていただき結果をご連絡下さい。

ａ：免疫力の変化と安全性を確認するため、以下の血液学的検査と血液生化学検査と免疫系の特殊検査をします。

承諾された60名はスクリーニングまたは0週及び・8週まで2週ごとに採血をします。

血液学的検査：WBC、RBC、Hb、Hct、Plt、白血球分画(好中球、リンパ球、単球、好塩基球、好酸球)

生化学検査：GOT、GPT、T-BIL、TP、ALB、CRP、Na、K、CL、BUN、Cr

特殊検査： CD16/CD56、NK細胞活性、CD4/CD8陽性細胞数、各種サイトカイン（IL-2、IL-4、IL-5、IL-6、IL-17、TNFα、IFN-α、IFN-γ、G-CSF、GM-CSF濃度）およびメタボロミクス解析用を採血する

ｂ：有害事象は、副作用など好ましくないすべての事象のことで、お薬との因果関係は問いません。

ｃ：被験者背景はアンケート調査を利用します。

ｄ：0週の採血については、スクリーニング検査時のデータを使用します。

ｅ：自覚症状(感冒症状を中心に身体症状)とバイタルサイン(体温・体重・必要時呼吸数/SpO_2、_浮腫時血圧測定)

### **7.1.1アンケート調査**

的確性の判断のため、試験責任医師又は試験分担医師は以下のアンケート調査検査を行い、選択基準を満たし、除外基準に抵触しない被験者を適格者とする。

アンケート項目

1. 被験者背景の調査（昨年の健診での結果を含む）
2. 自他覚症状(COVID19の症状を中心に身体所見をとる)
3. バイタルサイン(心拍数・血圧・体温・呼吸数/SpO_2_)
4. 併用薬、併用療法

### **7.1.2スクリーニング検査**

適格者が同意取得後、被験者情報を記録する。この時、アンケート調査記録を利用してもよい。なお、承諾の得られた60名のみ行う採血の検査項目は以下に記載の通りとする（1-3）。

該当参加者のみ

1. 血液学的検査(WBC、RBC、Hb、Hct、Plt、白血球分画：好中球、リンパ球、単球、好塩基球、好酸球)
2. 生化学検査(GOT、GPT、T-BIL、TP、ALB、BUN、CRP、Na、K、CL、Cr)
3. 特殊検査：、NK細胞活性、CD16/CD56、CD4/CD8陽性細胞数、各種サイトカイン（IL-2、IL-4、IL-5、IL-6、IL-17、TNFα、IFN-α、IFN-γ、G-CSF、GM-CSF濃度）およびメタボロミクス解析用

### **7.1.3被験者の情報**

同意取得時又はスクリーニング検査時に、以下の被験者情報を記録する。

　1) 同意取得日

　2) 被験者識別コード

　3) 被験者背景

　　・性別

　　・生年月（日については記録不要）

　　・年齢

　　・身長

　　・体重

・喫煙歴

　　・アルコール歴

　　・診断病日

　　・既往歴及び罹病期間

　　・合併症(併存症)・診断日

　　・基礎治療の内容と薬剤名

　　・他の服用薬剤の用法及び用量

　4) 現病歴

　　・COVID19または感冒症状の有無

　　・体温

　　・職場

### **7.1.4観察・検査・評価項目**

Visitごとの問診検査項目を以下に記す。

なお、0－8週の問診は、日記（ネット記録も含む）または診療録からも調査する。また、浮腫と血圧上昇がある場合は適宜、血清カリウムの測定ができる。

### **7.1.4.1．0週（Day0）**

1. 自覚症状(感冒症状を中心に身体症状)
2. バイタルサイン(体温・必要時呼吸数/SpO_2、_浮腫時血圧測定)
3. 有害事象の観察

以下該当患者のみ

1. 血液学的検査(WBC、RBC、Hb、Hct、Plt、白血球分画：好中球、リンパ球、単球、好塩基球、好酸球)
2. 生化学検査(GOT、GPT、T-BIL、TP、ALB、BUN、CRP、Na、K、CL、Cr)
3. 特殊検査： CD16/CD56、NK細胞活性、CD4/CD8陽性細胞数、各種サイトカイン（IL-2、IL-4、IL-5、IL-6、IL-17、TNFα、IFN-α、IFN-γ、G-CSF、GM-CSF濃度）およびメタボロミクス解析用

＊併用薬、併用療法の調査はアンケート調査、体重、血液検査、についてはスクリーニング時の検査値を利用してもよい。

### **7.1.4.2　2週（Day14）**

1. 自覚症状(感冒症状を中心に身体症状)
2. バイタルサイン(体温・体重・必要時呼吸数/SpO_2・_浮腫時血圧測定)
3. 有害事象の観察

以下該当患者のみ

1. 血液学的検査(WBC、RBC、Hb、Hct、Plt、白血球分画：好中球、リンパ球、単球、好塩基球、好酸球)
2. 生化学検査(GOT、GPT、T-BIL、TP、ALB、BUN、CRP、Na、K、CL、Cr)
3. 特殊検査： CD16/CD56、NK細胞活性、CD4/CD8陽性細胞数、各種サイトカイン（IL-2、IL-4、IL-5、IL-6、IL-17、TNFα、IFN-α、IFN-γ、G-CSF、GM-CSF濃度）およびメタボロミクス解析用

### **7.1.4.3　4週（Day28）**

1. 自覚症状(感冒症状を中心に身体症状)
2. バイタルサイン(体温・体重・必要時呼吸数/SpO_2・_浮腫時血圧測定)
3. 有害事象の観察

以下該当患者のみ

1. 血液学的検査(WBC、RBC、Hb、Hct、Plt、白血球分画：好中球、リンパ球、単球、好塩基球、好酸球)
2. 生化学検査(GOT、GPT、T-BIL、TP、ALB、BUN、CRP、Na、K、CL、Cr)
3. 特殊検査： CD16/CD56、NK細胞活性、CD4/CD8陽性細胞数、各種サイトカイン（IL-2、IL-4、IL-5、IL-6、IL-17、TNFα、IFN-α、IFN-γ、G-CSF、GM-CSF濃度）およびメタボロミクス解析用

### **7.1.4.4　6週（Day42）**

1. 自覚症状(感冒症状を中心に身体症状)
2. バイタルサイン(体温・体重・必要時呼吸数/SpO_2・_浮腫時血圧測定)
3. 有害事象の観察

以下該当患者のみ

1. 血液学的検査(WBC、RBC、Hb、Hct、Plt、白血球分画：好中球、リンパ球、単球、好塩基球、好酸球)
2. 生化学検査(GOT、GPT、T-BIL、TP、ALB、BUN、CRP、Na、K、CL、Cr)
3. 特殊検査： CD16/CD56、NK細胞活性、CD4/CD8陽性細胞数、各種サイトカイン（IL-2、IL-4、IL-5、IL-6、IL-17、TNFα、IFN-α、IFN-γ、G-CSF、GM-CSF濃度）およびメタボロミクス解析用

### **7.1.4.5　8週（Day56）**

1. 自覚症状(感冒症状を中心に身体症状)
2. バイタルサイン(体温・体重・必要時呼吸数/SpO_2・_浮腫時血圧測定)
3. 有害事象の観察

以下該当患者のみ

1. 血液学的検査(WBC、RBC、Hb、Hct、Plt、白血球分画：好中球、リンパ球、単球、好塩基球、好酸球)
2. 生化学検査(GOT、GPT、T-BIL、TP、ALB、BUN、CRP、Na、K、CL、Cr)
3. 特殊検査： CD16/CD56、NK細胞活性、CD4/CD8陽性細胞数、各種サイトカイン（IL-2、IL-4、IL-5、IL-6、IL-17、TNFα、IFN-α、IFN-γ、G-CSF、GM-CSF濃度）およびメタボロミクス解析用

### **7.1.4.6　12週（Day84）（追跡期間終了時）**

1. 自覚症状(感冒症状を中心に身体症状)
2. バイタルサイン(体温・体重・必要時呼吸数/SpO_2・_浮腫時血圧測定)
3. 有害事象の観察

### **7.1.4.7　中止時**

1. 自他覚症状(感冒症状を中心に身体症状)
2. バイタルサイン(体温、体重、心拍数血圧・体温・呼吸数/SpO_2_)
3. 有害事象の観察

# **有害事象発生時の取扱い**

## **有害事象の定義**

　有害事象とは試験薬が投与されたのちに生じる、好ましくない、あるいは意図しない徴候（臨床検査値の異常変動を含む）、症状または疾病のことであり、試験薬との因果関係を問わない。

## **有害事象発生時の被験者への対応**

　試験責任医師または試験分担医師は、有害事象を認めたときは、直ちに適切な処置を行うとともに、試験薬の投与を中止した場合や、有害事象に対する治療が必要となった場合には、被験者にその旨を伝える。

## **報告の対象となる有害事象**

試験薬が投与されてから試験薬投与終了後28日（Day72）までに発生したすべての有害事象は試験薬との因果関係の有無に関わらず報告し、有害事象が消失するか試験期間終了後（中止後）4週まで観察する。また、試験薬との因果関係があると判断された有害事象については試験期間終了時まですべて報告する。

## **有害事象発生時の報告手順**

上記期間に発生したすべての有害事象について、試験責任医師または試験分担医師は、カルテならびに症例報告書に齟齬なく記載する。

## **有害事象の評価に必要な記載内容**

1. 有害事象の名称
2. 発現日
3. 転帰日
4. 転帰：回復、消失、軽快、回復または消失したが後遺症あり、未回復、死亡、不明
5. 処置（試験薬の投与）：変更なし、中止、休薬、減量、増量、該当せず
6. その他の処置：なし、薬物治療、その他
7. 重篤度：非重篤、重篤
8. 重症度：軽度、中等度、高度
9. 試験薬との因果関係：関連あり、関連なし

### **有害事象の回復性と試験薬との因果関係**

　有害事象の消失とは、有害事象がない状態、又は投与前の状態への回復とする。有害事象における試験薬との因果関係の判定に際しては、被験者の全身状態、合併症、併用薬・併用療法、時間的関係を勘案して判断する。

# **重篤な有害事象発生時の取り扱い**

1. 疾病等について
   - 1. 疾病等（有害事象）の回復性と試験薬との因果関係

疾病等（有害事象）の消失とは、疾病等（有害事象）がない状態、又は投与前の状態への回復とする。疾病等（有害事象）における試験薬との因果関係の判定に際しては、被験者の全身状態、合併症、併用薬・併用療法、時間的関係を勘案して判断する。なお、因果関係の判断は「否定できる」、「否定できない」の2つの判定区分を用いる。

- - 1. 疾病等（有害事象）発生時の対応と報告
    2. 疾病等（有害事象）発生時の対応

試験責任医師または試験分担医師は、疾病等（有害事象）を認めたときは、直ちに適切な処置を行うとともに、カルテならびに症例報告書に齟齬なく記載する。また、試験薬の投与を中止した場合や、疾病等（有害事象）に対する治療が必要となった場合には、被験者にその旨を伝える。

- - 1. 疾病等（重篤な有害事象）発生時の対応

1) 試験責任医師又は試験分担医師は、疾病等（重篤な有害事象）が発現した場合、試験薬との因果関係を問わず適切な処置を行うとともに原因の究明につとめる。

2) 研究代表医師/研究事務局は、報告内容の緊急性、重要性、影響の程度などを判断し、必要に応じて登録の一時停止や参加施設への周知事項の緊急連絡などの対策を講ずる。

- - 1. 疾病等（重篤な有害事象）報告の手順

有害事象が発生し、研究責任医師が［研究代表医師への］報告を必要と判断した場合（重篤な有害事象）、下記の手順に従い当該有害事象情報を取り扱う。

1) 研究責任医師から［研究代表医師へ］の報告

研究責任医師は、当該有害事象情報を可能な限り速やかに実施医療機関の管理者［および研究代表医師］に報告する。報告は第1報（緊急報告）および第2報（詳細報告）とする。

2) 臨床研究審査委員会への報告

臨床研究法に基づき、研究責任医師［研究代表医師］が表1に従い臨床研究審査委員会への報告の必要性を判断した場合には、研究責任医師［研究代表医師］は「医薬品の疾病等報告書」（統一書式8）を作成し、臨床研究審査委員会に報告する。

［3) 研究代表医師による各実施医療機関への通知

研究代表医師は研究責任医師から入手した疾病等報告の内容を確認し、他の実施医療機関の研究責任医師に疾病等報告の情報を通知する。各研究責任医師はそれぞれの実施医療機関の管理者に当該有害事象情報を報告する。］

4) 追加情報の入手時の対応

当該有害事象が発生した臨床研究機関の研究責任医師は、当該事象に関する追加情報が得られた場合には、可能な限り速やかに実施医療機関の管理者［および研究代表医師］に追加報告を行う。当該追加情報の取扱いは2）、3）の手順に準ずる。

5) 定期報告

臨床研究機関の研究責任医師［研究代表医師］は、実施計画を厚生労働大臣に提出した日から起算して、１年ごとに、当該期間満了後２月以内に認定臨床研究審査委員会への定期報告を行う。実施医療機関の管理者に報告した上で「定期報告書」（統一書式5）を提出する。

6) 医薬品・医療機器等安全性情報報告制度に基づく対応

市販後の薬剤については医薬品・医療機器等安全性情報報告制度（医薬品、医療機器等の品質、有効性及び安全性の確保等に関する法律（昭和35年法律第145号）第68条の10第2項）に基づいて対応し、必要に応じて厚生労働省に報告する。

表1

| 予測可能性 | 疾病等の重篤性 | 報告先 | 報告期限 |
| --- | --- | --- | --- |
| 予期できない | 1. 死亡 2. 死亡につながるおそれ | 厚生労働大臣  臨床研究審査委員会 | 7日 |
| 予測できる | 1. 死亡 2. 死亡につながるおそれ | 臨床研究審査委員会 | 15日 |
| 予期できない | 1. 治療のために医療機関への入院又は入院期間の延長 2. 障害 3. 障害につながるおそれ 4. 上記に準じて重篤 5. 後世代における先天性の疾病又は異常 | 厚生労働大臣  臨床研究審査委員会 | 15日 |

## **重篤な有害事象の定義**

疾病等の定義

疾病等とは、特定臨床研究の実施に起因するものと疑われる疾病、障害若しくは死亡または感染症に加え、臨床検査値の異常や諸症状を含む（特定臨床研究との因果関係が否定できない有害事象）。なお、臨床研究審査委員会への報告の必要な疾病等は、「疾病等（重篤な有害事象）報告の手順」に従い報告を行う。

重篤な有害事象とは、次のいずれかに該当するものとする。

（1） 死亡

（2） 死亡につながるおそれのあるもの

（3） 障害（日常生活に支障をきたす程度の機能不全の発現）

（4） 障害につながるおそれのあるもの

（5） 治療のために病院又は診療所への入院又は入院期間の延長が必要とされるもの

（6） （1）～（5）までに掲げる症例に準じて重篤であるもの

（7） 後世代における先天性の疾病又は異常

なお、（5）の「入院」には、再検査、追跡調査のための入院又は入院期間の延長、及び試験前より予定していた療法又は検査を試験中に実施すること、COVIDの治療を目的とした入院と予定手術や検査等は含まれない。（ただし、その入院中に新たに発生したものは有害事象として取扱う。）

## **報告の対象となる重篤な有害事象**

試験期間中の全ての重篤な有害事象、および試験終了（中止）後に試験薬との関連性が疑われる重篤な有害事象について実施医療機関の管理者に報告する。

(非重篤な有害事象)

非重篤な有害事象とは、「重篤」と判定されたもの以外の有害事象とし、その判定は試験責任医師または試験分担医師が行う。

# **評価項目**

## **主要評価項目**

症状を有するCOVID-19のPCR陽性者数

（症状とは37.5度以上の発熱、倦怠感、咳嗽、咽頭痛、嘔吐、下痢、呼吸困難、味覚・嗅覚異常などの感冒様症状を指す）。

【主要評価項目の設定根拠】

本薬剤がCOVID-19の感染予防ができるかを検討する。ただしCOVID-19は感染後無発症例が存在する。現時点（2020年5月15日現在）日本ではPCRが無症状の方に自由に検査できる状態でないため、COVID-19 の感染者が来院される医療機関従事者における感染および感染後の発症率に関する確固たるデータはない。医療機関従事者の感染後の発症を取集するため、発症症例に加えPCR検査の陽性を確認する。

## **副次評価項目**

**【有効性の副次評価項目】**

1. 感染（自己申告に基づく）から発症までの期間
2. 症状出現からPCR陰性までの期間
3. 症状出現から症状の改善までの期間
4. 酸素吸入を有する入院（重症）の有無
5. 重篤（機械換気を要する、ショックバイタル、肺以外の臓器不全にてICU 管理が必要）

**【安全性の副次評価項目】**

有害事象の発生頻度

【有効性の副次評価項目の設定根拠】

1. COVID-19は感染後無発症例が存在する。医療機関での検査において感染日の特定は難しく自己申告における感染日から発症の期間および感染日から症状発現までの期間の検討のため設定した。
2. 症状（かつPCR陽性）の発症時がCOVID-19の発症(最大活性時点)と考えられるため、その期間（PCR陰性までも含む）を検討する。
3. 同じく症状（かつPCR陽性）の持続期間への影響を検討する
4. 本薬剤がCOVID-19の重症化抑制効果があるかを指標にする事ができるかを検討する。
5. 本薬剤がCOVID-19の重篤化抑制効果があるかを指標にする事ができるかを検討する。

【安全性の副次評価項目の設定根拠】

本薬剤は、市販されているため、頻度としては甘草による偽アルドステロン症が主なもので、重篤なものとして肝機能障害や間質性肺炎が考えられる。また、CTCAEやMedDRAを用いて用語の統一を行う。

## **探索的評価項目**

補中益気湯の有効時のメカニズムを探索するために、特殊検査として以下を内服期間中経時的に行う。

自然免疫検査（当日）： CD16/CD56、NT細胞活性、CD4/CD8陽性細胞数

獲得免疫検査（後日）TNFα、ILシリーズ（IL-2、IL-4、IL-5、IL-6、IL-17濃度など）およびメタボロミクス解析（選択検体のみ）

【探索的評価項目の設定根拠】

補中益気湯は基礎的及び臨床で自然免疫系に作用が報告されている。また、獲得免疫系への影響を調べる。

# **統計学的事項**

　本試験の統計解析計画の概要を以下にまとめた。なお、統計解析計画の詳細は、統計解析計画書に記載する。統計解析計画書において本試験実施計画書の概要を修正することがあるが、主要評価項目の定義や解析方法が変更される場合には、本試験実施計画書を改訂する。

## **解析対象集団**

### **最大の解析対象集団** **(full analysis set：FAS)**

　本試験に登録され1 回以上試験薬を投与され、有効性データがあるすべての被験者を最大の解析対象集団 (FAS) とする。ただし、ベースラインのデータが取得できない被験者及び、重大な試験実施計画書違反 (同意未取得、契約期間外の登録等) の被験者については除外する。

### **試験実施計画書に適合した対象集団 (per protocol set：PPS)**

FAS の被験者のうち、以下の基準により特徴付けられる対象集団について記載する。
(1) 事前に定められた最低限の試験治療規定を完了していること、(2) 主要変数の測定値が利用可能であること、(3) 登録基準違反などの重大な試験実施計画書違反がないこと。

FAS から、試験方法や併用療法など試験実施計画書の規定に対して、以下の重大な違反があった症例を除いた被験者とする。

選択基準違反

除外基準違反

併用禁止薬違反

コンプライアンス不良 (80% 未満の内服の時)

### **安全性解析対象集団**

本試験に登録され、少なくとも 1 度は試験薬を投与された症例を解析の対象とし、実際に投与された試験治療を群とする。

## **目標症例数と設定根拠**

解析対象例数：6000例

【設定根拠】

本試験では、COVID-19による発症抑制の予防作用の検討を行う。試験薬投薬前の感染割合は0%、その後のCOVID-19の発症割合を2-3%、そのうちの70%-80%が発症すると仮定した。試験薬による薬剤効果を50%と期待して、プラセボの発症割合を1.4%-2.4%と想定、試験薬による発症割合を0.7%-1.2%と想定し、プラセボを2%、試験薬を1%として症例数を算出した。検証する頻度が低く、症例数が多くなってしまうため、9回の中間解析と1回の最終解析の設定をした。Haybittle-Peto法^＊1~*3 ）^にて最終解析時点でα=0.05、検出力80% の条件で4,986例となる脱落率 を10-20% 程度と想定しと見積り症例数を算出し、各中間解析で 600例ずつ、最終 6,000例の症例数を設定した。

^*1) The SEQDESIGN Procedure SAS/STAT User’s Guide^

^https://support.sas.com/documentation/onlinedoc/stat/123/seqdesign.pdf^

^*2) Haybittle, J. L. (1971). "epeated Assessment of Results in Clinical Trials of Cancer Treatment." British Journal of Radiology^ **^44^**^:793–797.^

^*3) Peto, R., Pike, M. C., Armitage, P., Breslow, N. E., Cox, D. R., Howard, S. V., Mantel, N., McPherson, K., Peto, J., and Smith, P. G. (1976). "Design and Analysis of Randomized Clinical Trials Requiring Prolonged Observation of Each Patient: I. Introduction and Design." British Journal of Cancer^ **^34^**^:585–612.^

| **Step** | | **合計症例数**  **（小数症例数）** | **脱落率を考慮した**  **合計症例数**  **（小数症例数）** | **目標症例数** | **各群の**  **目標症例数** |
| --- | --- | --- | --- | --- | --- |
|  | **比率** | **N** | **N**  **脱落率**  **10%～20%** | **N** | **N** |
| 1 | 0.1 | 498.583 | 554.0 ～ 692.5 | 600 | 300 |
| 2 | 0.2 | 997.167 | 1108.0 ～ 1385.0 | 1,200 | 600 |
| 3 | 0.3 | 1495.750 | 1661.9 ～ 2077.4 | 1,800 | 900 |
| 4 | 0.4 | 1994.334 | 2215.9 ～ 2769.9 | 2,400 | 1,200 |
| 5 | 0.5 | 2492.917 | 2769.9 ～ 3462.4 | 3,000 | 1,500 |
| 6 | 0.6 | 2991.500 | 3323.9 ～ 4154.9 | 3,600 | 1,800 |
| 7 | 0.7 | 3490.084 | 3877.9 ～ 4847.3 | 4,200 | 2,100 |
| 8 | 0.8 | 3988.667 | 4431.9 ～ 5539.8 | 4,800 | 2,400 |
| 9 | 0.9 | 4487.250 | 4985.8 ～ 6232.3 | 5,400 | 2,700 |
| 10 | 1.0 | 4985.834 | 5539.8 ～ 6924.8 | 6,000 | 3,000 |

## **症例の取り扱い**

原則として登録された症例については、試験調整医師および統計専門家が協議の上、症例の取り扱いを決定する。新たな問題が起こった場合の症例の取り扱いについても、試験調整医師および統計専門家が、協議の上、決定する。

## **データの取り扱い**

データ集計・解析時におけるデータの取り扱いについては、原則として以下に示す通りとする。疑義が生じた場合は、統計専門家と試験調整医師が協議の上決定する。

欠測値に対しては、必要に応じて補完を行う。詳細については、統計解析計画書に記載する。

## **統計解析項目および解析計画**

全ての症例において試験薬の投与が終了し、データが固定された後に解析を行う。全ての有効性評価において、最大の解析対象集団 (FAS) における解析を主解析とし、参考として試験実施計画書に合致した解析対象集団 (PPS) における解析を行う。安全性の解析は、安全性解析対象集団における解析を実施する。

　統計解析の詳細はデータ固定前に別途作成する統計解析計画書に規定する。

### **被験者背景の解析**

　各解析対象集団における被験者背景データの分布及び要約統計量を群ごとに算出する。名義変数については、カテゴリの頻度及び割合を群ごとに示す。連続変数については要約統計量 [例数、平均値、標準偏差、最小値、中央値、最大値]IQR(25%、75%点) を群ごとに算出する。群間の比較には、名義変数については、カイ 2 乗検定またはFisher の直接確率計算法、順序変数については、Wilcoxon の順位和検定、連続変数については t 検定を用いる。有意水準は両側 5% とする。

### **主要評価項目の解析**

主たる解析項目として、症状を有するCOVID-19のPCR陽性者数の有無割合及びその信頼区間を求める。（症状とは37.5度以上の発熱、倦怠感、咳嗽、咽頭痛、嘔吐、下痢、呼吸困難、嗅覚・味覚異常など感冒様症状を指す）。

### 副次評価項目の**解析**

　主たる解析結果を補足する考察を行う目的で有効性の副次評価項目の解析を行う。

### **安全性の解析**

安全性の評価項目は、有害事象の発生頻度であり、評価項目について集計表を作成する。

### **中間解析**

　本試験における主要評価は安全性評価と密接に基づく項目である。独立データモニタリング委員会での重篤性の判断や想定感染率の違いなどの確認を実施したことによる判断には有意水準αの消費は考慮しない。症例数の設定時に、Peto法により9回の中間解析と1回の最終解析において最終解析での有意水準α=0.05 と検出力1-β=0.80 を確保する症例数を算出した。各ステップにおける境界値を以下に示す。

| **Step** | **情報レベル** | | | **対立** | | **境界値** | | **停止確率** | |
| --- | --- | --- | --- | --- | --- | --- | --- | --- | --- |
|  |  |  |  | **参照** | | **下限** | **上限** |  |  |
|  | **比率** | **実測値** | **N** | **下限** | **上限** | **α** | **α** | **α** | **1-β** |
| 1 | 0.1 | 1.640 | 498.583 | -0.9006 | 0.9006 | -3.0000 | 3.0000 | 0.0027 | 0.0179 |
| 2 | 0.2 | 3.279 | 997.167 | -1.2736 | 1.2736 | -3.0000 | 3.0000 | 0.0049 | 0.0511 |
| 3 | 0.3 | 4.919 | 1495.750 | -1.5599 | 1.5599 | -3.0000 | 3.0000 | 0.0067 | 0.0955 |
| 4 | 0.4 | 6.559 | 1994.334 | -1.8012 | 1.8012 | -3.0000 | 3.0000 | 0.0083 | 0.1483 |
| 5 | 0.5 | 8.199 | 2492.917 | -2.0138 | 2.0138 | -3.0000 | 3.0000 | 0.0096 | 0.2068 |
| 6 | 0.6 | 9.838 | 2991.500 | -2.2060 | 2.2060 | -3.0000 | 3.0000 | 0.0107 | 0.2688 |
| 7 | 0.7 | 11.478 | 3490.084 | -2.3828 | 2.3828 | -3.0000 | 3.0000 | 0.0118 | 0.3321 |
| 8 | 0.8 | 13.118 | 3988.667 | -2.5473 | 2.5473 | -3.0000 | 3.0000 | 0.0127 | 0.3952 |
| 9 | 0.9 | 14.758 | 4487.250 | -2.7018 | 2.7018 | -3.0000 | 3.0000 | 0.0136 | 0.4567 |
| 10 | 1 | 16.397 | 4985.834 | -2.8479 | 2.8479 | -2.0213 | 2.0213 | 0.0500 | 0.8001 |

## **11.6. 独立データモニタリング委員会**

　本試験では独立データモニタリング委員会を設置する。独立データモニタリング委員会は自ら試験を実施する者と独立した機関として設立され、本試験とは独立した立場である 2人以上の専門家による委員で構成される。独立データモニタリング委員会は、患者の安全性を確保することを目的に、必要に応じて、被験治療及び対照における有害事象発現率の比較、重篤な有害事象に関する詳細な検討等の安全性モニタリングを行う。ときにその結果を踏まえて有害事象のリスクを軽減する為に、組入れ基準の変更等の試験デザインの変更を勧告すること、あるいは試験の継続の可否を判断することもある。

## **最終解析**

追跡期間終了後、データが得られ症例が固定された後に解析を行う。統計解析責任者が「解析報告書」をまとめ、試験調整医師及び試験責任医師に提出する。

# **試験実施計画書の遵守および逸脱**

1. 試験責任医師又は試験分担医師は、試験実施計画書から逸脱した行為を理由のいかんによらずすべて記録する。
2. 被験者の緊急の危機を回避するためその他医療上やむを得ない理由により実施計画書から逸脱した場合、試験責任医師は、逸脱の内容及びその理由を記載した文書を実施医療機関の管理者に直ちに提出するとともに、当該文書の内容を実施医療機関の管理者を経由して認定臨床研究審査委員会に速やかに報告する。

# **試験実施計画書、症例報告書又は解析計画に関する変更**

## **試験実施計画書および症例報告書の改訂**

試験実施計画書及び症例報告書を改訂する場合には、以下の手順により行う。

1. 試験責任医師は試験実施計画書改訂版及び症例報告書改訂版を速やかに実施医療機関の管理者に提出し、実施医療機関の管理者を経由して速やかに認定臨床研究審査委員会の承認を得る。
2. 認定臨床研究審査委員会の意見に基づく実施医療機関の管理者の指示が研究代表者および試験調整委員会の許容できる範囲内で、試験実施計画書及び症例報告書用紙を修正する場合も同様の手順とする。

＜多施設共同試験の場合＞

1. 研究代表者および試験調整委員会は、改訂が必要と認めた場合には、試験責任医師に試験実施計画書改訂案、症例報告書改訂案及び、最新の添付文書、その他必要な資料・情報を提供する。
2. 研究代表者および試験調整委員会は、試験責任医師が前項により提供された試験実施計画書改訂案等の資料・情報を十分検討し、研究代表者および試験調整委員会と協議するのに必要な時間を試験責任医師に与える。
3. 研究代表者および試験調整委員会と協議した後、試験責任医師は試験実施計画書改訂版及び症例報告書用紙改訂を速やかに実施医療機関の管理者に提出し、実施医療機関の管理者を経由して速やかに認定臨床研究審査委員会の承認を得る。
4. 認定臨床研究審査委員会の意見に基づく実施医療機関の管理者の指示が研究代表者および試験調整委員会の許容できる範囲内で、試験実施計画書及び症例報告書用紙を修正する場合も同様の手順とする。

## **統計解析計画の変更**

　統計解析責任者は、統計解析計画書の内容を変更した場合、変更内容をすべて本試験の統計解析報告書に記載する。なお、統計解析計画書の変更は、その経緯を記録に残す。

# **試験の中止、中断または終了**

## **試験全体での中止または中断の基準**

研究代表者および試験調整委員会は、以下の情報が得られ、試験全体の続行が困難であると考えられる時には、試験責任医師と試験全体の中止又は中断について協議のうえ、決定する。

1. 予期できない重篤な副作用の発生
2. 予期できる重篤な副作用の発生件数、発生頻度、発生条件等の発生傾向がインタビューフォーム・添付文書から予測できないことを示す情報
3. 重篤な有害事象のうち因果関係がないと判断されていたが、その後発生数、発生頻度、発生条件等の発生傾向から因果関係が否定できないと判断される情報
4. 副作用の発生数、発生頻度、発生条件等の発生傾向が著しく変化したことを示す研究報告
5. がんその他の重大な疾病、障害もしくは死亡が発生するおそれがあることを示す研究報告
6. 当該治験で有効性が認められないことを示唆する情報
7. 試験の対象となる疾患に対して効能もしくは効果を有していないことを示す情報
8. 試験薬と同一成分を含む市販医薬品について、製造、輸入又は販売の中止、回収、廃棄その他の保健衛生上の危害の発生又は拡大を防止するための措置の実施の情報

## **試験全体での中止又は中断する場合の手続き**

　試験責任医師は試験全体を中止又は中断する場合には、認定臨床研究審査委員会および実施医療機関の管理者にその旨とその理由を詳細に速やかに文書で通知する。また、投与中の被験者に対して速やかにその旨を伝え、適切な治療への変更等の適切な処理を行うものとする。

＜多施設共同試験の場合＞

　研究代表者または試験調整委員会は、他の試験責任医師と協議のうえ試験全体を中止又は中断する場合には、実施医療機関の管理者にその旨とその理由を詳細に速やかに文書で通知する。また、投与中の被験者に対して速やかにその旨を伝え、適切な治療への変更等の適切な処理を行うものとする。

## **個々の実施医療機関での試験を中止または中断する場合の手続き**

　試験責任医師は、試験を中止又は中断した場合には、実施医療機関の管理者に速やかにその旨を文書で通知するとともに、中止又は中断について文書で詳細に説明する。

研究代表者は、試験責任医師が試験を中止又は中断した旨を通知してきた場合には、中止又は中断について詳細に説明された文書により当該試験に関与するすべての試験責任医師に速やかに文書で通知する。

## **試験の終了**

　試験責任医師は、試験終了後、実施医療機関の管理者に試験が終了した旨を文書で通知し、試験結果の概要を文書で報告する。

# **データマネジメント**

## **データ登録の方法及び管理方法**

1. 症例報告書（Case Report Form ; CRF）はICH-GCPの要件に対応したソフトウエアを用いた電子症例報告書（eCRF）を使用する。
2. 電子症例報告書(eCRF)によるデータの収集
3. 研究責任医師又は研究分担医師はeCRFを作成する。研究協力者は原資料（原データ）を転記しeCRFを作成する。記載内容の変更、修正又は追記に当たっては、eCRFを作成したソフトウエア上で行い、全て電子情報として記録する。研究責任医師はeCRFに入力される全てのデータの正確性と信頼性について責任を負う。
4. 電子症例報告書の保管

電子症例報告書（eCRF）の原本は、データ固定後にシステム上の電子症例報告書（写）を電子媒体に保管したものとする。最終的には、eCRFの原本は研究責任医師に提供する。

1. データ固定

試験責任医師が確認後、データ固定を行う。

## **症例報告書に直接記載され、かつ原資料(原データ)と解すべき資料の特定**

本試験においては、以下の文書などを原資料(原データ)とする。

1. 被験者の同意及び被験者への情報提供に関する記録

診療録、看護記録、臨床検査データ及び画像検査フィルム等症例報告書作成の基となった記録。なお、電子カルテに格納されたデータも原資料とみなす。

1. 試験薬投与に関する記録
2. 本試験に関連する指針上必要な試験に係る文書又は記録

症例報告書に記載されたデータのうち、以下に示す項目は症例報告書の記載をもって原資料(原データ)とする。ただし、診療録等に記載のある場合は、当該診療録等を原資料(原データ)とみなす。

1. 併用薬・併用療法の目的
2. 有害事象の程度、転帰(追跡調査時の結果を含む)、重篤度、本試験薬との因果関係の判定及び判定根拠
3. 被験者の試験中止理由
4. 試験責任医師又は試験分担医師のコメント

# **原資料及びその他の記録の保存**

　研究機関の長および試験責任医師は、当該研究に係る情報等について、少なくとも当該研究の終了後5年を経過した日または当該研究の結果公表後5年を経過した日のいずれか遅い日まで、適切に保存しなければならない。

# **試料等の保存及び他機関等の試料等の利用**

# 試験に関する採血により得た血清等は、名古屋市立大学大学院薬学研究科牧野教室（牧野利明教授）にて、一部解析後に保存して、COVID19に関連する検査のみに使用する。保存は、それ以外の用途に使わないこととし管理者（牧野利明教授）が責任をもって管理する。

# 提供をする試料は個人情報とはリンクできない番号を付して牧野教室に提出する。提供する試験参加者には、破棄の希望があるかを確認するインフォームドコンセントも併せて行う。

# **原資料の直接閲覧**

　試験実施医療機関の管理者及び試験責任医師は、モニタリング、監査及び認定臨床研究審査委員会又は規制当局による調査の際に、原資料等すべての記録を閲覧できることを保証する。試験が適切に実施されていること及びデータの信頼性が十分に確保されていることを確認する。

# **試験の品質管理及び品質保証**

## **品質管理**

## 本試験が安全に、かつ実施計画書に従って実施されているか、データが正確に集積されているかを確認する目的でモニタリングを行う。モニタリングは、「臨床試験のモニタリングと監査に関するガイドライン」に基づいて行う。定期的にデータセンターに集積される症例報告書の記入データに基づいて中央モニタリングを実施する。また、リスクに基づき、施設における品質管理（オンサイト・オフサイトモニタリング）を別途定めるモニタリング手順書に従って行う。

モニタリングに従事する者は、当該モニタリングの結果を研究責任者に報告しなければならない。また、業務上知りえた情報を正当な理由なく漏らしてはならない。その業務に従事しなくなった後も同様とする。

## **品質保証**

研究代表者は本試験の品質保証の為に、必要に応じて監査は実施しない。

# **倫理**

　本試験は臨床研究法およびヘルシンキ宣言を遵守して実施する。

当該臨床研究において、臨床研究対象者に生じる利益は当該薬による予防効果であり、その際に生じる副作用による不利益は、定期的な日記により早期に把握するようにして、迅速な対応をすることで最小化する。このような体制を組むことで、倫理的にも人道的な配慮をする。

# **被験者の秘密の保全**

試験責任医師は被験者の個人情報が守られていることを確認しなければならない。

1. 症例報告書では被験者の識別は固有の被験者識別番号のみで行う。
2. 被験者の個人情報が記載された同意文書などの書類は試験担当医師が機密文書として扱う。

# **認定臨床研究審査委員会**

　本試験の実施に先立ち、実施医療機関の認定臨床研究審査委員会は、本試験の倫理的、科学的及び医学的妥当性を審査する。本試験は、認定臨床研究審査委員会の承認を得た後に実施する。また、認定臨床研究審査委員会は厚生労働省令で定めるところにより，定期的に特定臨床研究の実施状況について報告を受け，当該特定臨床研究の継続の適否について意見を述べる．

# **健康被害補償及び保険**

## **健康被害の補償**

健康被害については、責任医師等はその回復に努め適切な医療を提供するものとする。本研究は予防という適応外で使用して行うが既に市販されている薬ある。そのお薬による健康被害の治療は、通常の診療と同様に医療関係者のみなさんの健康保険を用いて行う場合もある。明らかに本研究が原因となった場合の補償に備え、臨床研究保険に入る。

## **臨床研究保険（補償保険）への加入**

被験者の健康被害への補償責任に備え、試験責任医師および試験分担医師は臨床研究保険（補償保険）に加入するが、COVID19感染自体には適応されない。

## **賠償保険への加入**

賠償責任に備え、試験責任医師および試験分担医師は賠償責任保険に加入する。

# **金銭の支払い**

　試験薬（補中益気湯）および採血検査の費用は研究費により賄われる。試験参加に伴って特別に費用負担が増加することはなく、原疾患、他の合併症に対する検査・処置・治療は健康保険の範囲内で行われる。

# **研究資金および利益の衝突**

本試験は、実施主体である一般社団法人日本東洋医学会であり医学・薬学における学術の振興に寄与し、その成果を広く社会に還元することで、国民の健康と福祉に貢献することを目的とする。本研究(試験)計画は、千葉大学和漢診療学の並木隆雄（研究代表者）が代行し、奨学寄付金（日本東洋医学会）で行われる。大杉製薬株式会社からは試験薬(実薬と偽薬)の現物支給である。試験薬に関する情報は提供するが、試験の実施、解析、報告に係わることはない。

本研究(試験)は千葉大学の主任研究者(試験責任医師)により公正に行われる。また、研究資金については、資金計画書に基づいて運用される。本研究の利害関係については、千葉大学医学部附属病院利益相反委員会の承認を得た上で、本学附属病院「臨床研究に関する利益相反ポリシー」に従い、適切に利益相反のマネジメントを行い、また、当該研究(試験)の経過を定期的に利益相反委員会へ報告等行うことにより、本研究の利害関係についての公正性を保つ。

　本試験の研究代表者（および各施設の試験責任医師ならびに試験分担医師）には開示すべき利益相反はない。

# **研究に関する情報公開**

　試験責任医師は、当該臨床試験について臨床研究実施計画・研究概要公開システム（JRCT）の公開データベースに 当該研究の概要をその実施に先立って登録し、実施計画書の変更及び試験の進捗に応じて適宜更新する。また、臨床試験を終了したときは、遅滞なく、当該臨床試験の結果を登録する。

# **結果の公表**

## **公表の方法**

試験責任医師は、試験を終了したときは、遅滞なく、被験者等及びその関係者の人権又は研究者等及びその関係者の権利利益の保護のために必要な措置を講じた上で、当該試験の結果を公表する。結果の公表方法としては、学会発表や論文掲載、公開データベースへの登録（試験実施計画書、統計解析計画書試験実施計画書とは別に作成されている場合）及び同意説明文書、総括報告書等を含む）。

## **公表についての取り決め**

主たる公表論文は英文誌に投稿する。

研究代表者または試験調整医師による、研究のエンドポイントの解析結果を含まない、研究の紹介目的の学会・論文（総説）発表や、登録終了後の、患者背景の分布や安全性データの学会・論文発表は試験調整員会の了承を得て行うことができる。これらに該当しない、主たる解析と最終解析以外の発表については、事前に独立データモニタリング委員会の承認を得た場合を除いて行わない。原則として、研究結果の主たる公表論文（primary endpointの結果を初めて公表する論文）の著者は筆頭を試験調整医師とし、以下、研究代表者、統計解析担当（公表のための解析を行った時点での担当者 1 名）する。それ以下は、論文の投稿規定による制限に従って、登録数の多い順に貢献度の高かった施設研究者を施設毎に選んで共著者とし、最終著者は研究グループ代表（又は研究代表者）とする。試験調整事務局の担当者を著者に含めるかどうかは、貢献度に応じて研究代表者が決定する。主たる公表論文以外の論文（Secondary endpoints に関する論文、副次的解析の論文など）の著者は、研究代表者が試験調整委員会の了承を得て決定する。

すべての共著者は、投稿前に論文内容をレビューし、発表内容に合意した者のみとする。内容に関して、議論にても合意が得られない場合、研究代表者は試験調整委員会の了承を得て、その研究者を共著者に含めないことができる。

主たる学会発表（primary endpoint の結果の初めての学会発表）の筆頭演者は原則として試験調整医師とする。その他の学会発表は複数回に及ぶ可能性があるため、試験調整医師、研究代表者、登録の多い施設の研究責任者の中から、持ち回りで発表を行うこととする。

発表者は研究代表者の了承を得て決定する。ただし、学会発表に際しては、発表準備および発表内容について試験調整委員会が責任を持ち、原則として統計解析担当者との連絡は試験調整事務局が行う。試験調整委員会以外の発表者が、試験調整委員会と統計解析責任者、データセンターの了承なく、直接データセンターから集計・解析結果を受け取ることはできない。

# **参考資料・文献リスト**

＜参考資料・引用文献を記入してください＞

1. Chen Q, Liang M, et al. [Mental health care for medical staff in China during the COVID-19 outbreak.](https://pubmed.ncbi.nlm.nih.gov/32085839/?from_term=covid+19+psychiatry&from_pos=1) Lancet Psychiatry. 2020 Apr;7(4): e15-e16
2. Dalton L, Rapa E, Stein A.Dalton L, et al：[Protecting the psychological health of children through effective communication about COVID-19.](https://pubmed.ncbi.nlm.nih.gov/32243784/?from_term=covid+19+psychiatry&from_page=7&from_pos=1) Lancet Child Adolesc Health. 2020 Mar 31: S2352-4642(20)30097-3
3. 中華人民共和国国家衛生健康委員会弁公庁中国国家中医薬管理局弁公室

新型コロナウイルス肺炎診療ガイドライン（試行第7版）2020年3月3日

1. WHO: Report of the WHO-China Joint Mission Coronavirus Disease 2019 (COVID-19)
2. Tatsumi K, Shinozuka N, et al: **Hochuekkito** improves systemic inflammation and nutritional status in elderly patients with chronic obstructive pulmonary disease. J Am Geriatr Soc. 2009; 57: 169–70.
3. Cho S, Hong T et al: Evaluation of Immunological Effects of **Hochu-Ekki-To** (TJ-41) Prophylactic Administration in Mice Am J Chin Med 32 (2), 235-43 2004
4. Satoh N, Sakai S et al.：[A randomized double blind placebo-controlled clinical trial of **Hochuekkito**, a traditional herbal medicine, in the treatment of elderly patients with weakness N of one and responder restricted design.](https://pubmed.ncbi.nlm.nih.gov/16121514/?from_term=Hochuekkito+Terasawa+&from_pos=1) Phytomedicine. 2005 Aug;12(8):549-54.
5. Mori K, Kido T, Daikuhara H, et al：Effect of **Hochu-ekki-to**（TJ─41）, a Japanese herbal medicine, on the survival of mice infected with influenza virus. Antiviral Res, 1999；44：103〜111
